# ASSOCIATIONS OF RELATIVE INTENSITY OF PHYSICAL ACTIVITY WITH INCIDENT CARDIOVASCULAR OUTCOMES AND MORTALITY

**DOI:** 10.1093/geroni/igad104.1172

**Published:** 2023-12-21

**Authors:** Benjamin Schumacher, Michael LaMonte, Chongzhi Di, Humberto Parada Jr., Steven Hooker, John Bellettiere, Eleanor Simonsick, Andrea LaCroix

**Affiliations:** University of Pittsburgh School of Public Health, Pittsburgh, Pennsylvania, United States; University at Buffalo - SUNY, Buffalo, New York, United States; Fred Hutchinson Cancer Center, Seattle, Washington, United States; San Diego State University, San Diego, California, United States; San Diego State University, San Diego, California, United States; University of California, San Diego, San Diego, California, United States; NIA IRP, Baltimore, Maryland, United States; University of California, San Diego, San Diego, California, United States

## Abstract

For an older individual, the energy expended (absolute intensity) during a specific activity (e.g., casual walk) may be closer to their maximal effort (higher relative effort) than for a younger individual. We compared the associations of relative and absolute intensity of physical activity (PA) with all-cause mortality and incident major cardiovascular disease (CVD). Accelerometer-measured PA in the Objective Physical Activity and Cardiovascular Health (OPACH) Study (mean age 78.5±6.7) was used to estimate daily hours of absolute light intensity PA (LPA) and moderate-to-vigorous PA (MVPA). Accelerometer-estimated metabolic equivalents (METs) in each 15-second epoch were divided by two maximal MET capacity estimates. These percent maximal effort metrics were categorized and aggregated into daily hours of relative LPA and MVPA. Cox proportional hazards models estimated the associations of a one-hour daily increase in absolute and relative PA with the two outcomes. On each PA measurement scale, an increase in either intensity category reduced the risk of both outcomes. A one-hour increase in absolute LPA reduced the risks of both outcomes by 12%, and a one-hour increase in absolute MPVA reduced the risk of death and CVD by 45% and 27%, respectively. On the relative scale, LPA was more strongly associated with both outcomes than MVPA. Increasing absolute MVPA was more strongly associated with the outcomes than increasing relative MVPA. The PA intensity paradigm should keep shifting towards recommendation of more movement, regardless of intensity, and placing greater emphasis on relative light intensity activities to reduce the risks of death and incident major CVD.

